# The Chromosome 9p21.3 Coronary Heart Disease Risk Allele Is Associated with Altered Gene Expression in Normal Heart and Vascular Tissues

**DOI:** 10.1371/journal.pone.0039574

**Published:** 2012-06-29

**Authors:** Anna P. Pilbrow, Lasse Folkersen, John F. Pearson, Chris M. Brown, Les McNoe, Nancy M. Wang, Wendy E. Sweet, W. H. Wilson Tang, Michael A. Black, Richard W. Troughton, A. Mark Richards, Anders Franco-Cereceda, Anders Gabrielsen, Per Eriksson, Christine S. Moravec, Vicky A. Cameron

**Affiliations:** 1 Christchurch Cardioendocrine Research Group, Department of Medicine, University of Otago Christchurch, Christchurch, New Zealand; 2 Department of Medicine, Karolinska Institutet, Stockholm, Sweden; 3 Department of Public Health and General Practice, University of Otago Christchurch, Christchurch, New Zealand; 4 Department of Biochemistry, University of Otago, Dunedin, New Zealand; 5 Department of Cardiovascular Medicine, Kaufman Center for Heart Failure, Cleveland Clinic, Cleveland, Ohio, United States of America; 6 Department of Molecular Medicine and Surgery, Karolinska Institutet, Stockholm, Sweden; Johns Hopkins University, United States of America

## Abstract

Genome-wide association studies have identified a coronary artery disease (CAD) risk locus in a non-coding region at 9p21.3, the nearest genes being *CDKN2A* and *CDKN2B*. To understand the pathways by which this locus might influence CAD susceptibility, we investigated associations between the 9p21.3 risk genotype and global gene expression in heart tissue from donors with no diagnosed heart disease (n = 108, predominant cause of death, cerebral vascular accident) and in carotid plaque (n = 106), aorta (n = 104) and mammary artery (n = 88) tissues from heart valve and carotid endarterectomy patients. Genotyping was performed with Taqman assays and Illumina arrays, and gene expression profiles generated with Affymetrix microarrays. Associations were analyzed with an additive genetic model. In heart tissue, 46 genes were putatively altered in association with the 9p21.3 risk allele (70% down-regulated, fold-change >1.1 per allele, p<0.05 adjusted for age, gender, ethnicity, cause of death). These genes were enriched for biomarkers of myocardial infarction (p = 1.53×10^−9^), response to wounding (p = 2.65×10^−10^) and inflammatory processes (p<1.97×10^−7^). Among the top 10 most down-regulated genes, 7 genes shared a set of transcription factor binding sites within conserved promoter regions (p<1.14×10^−5^), suggesting they may be co-regulated. Canonical pathway modelling of the most differentially expressed transcripts across all tissues (154 genes, 60% down-regulated, fold-change >1.1 per allele, p<0.01) showed that 75% of the genes could be transcriptionally regulated through the cell cycle G1 phase progression pathway (p<1.08×10^−258^), in which *CDKN2A* and *CDKN2B* play a regulatory role. These data suggest that the cell cycle G1 phase progression pathway is activated in individuals with the 9p21.3 risk allele. This may contribute to a proliferative phenotype that promotes adverse cardiac hypertrophy and vascular remodeling, leading to an increased CAD risk.

## Introduction

Coronary artery disease (CAD) is a leading cause of mortality and morbidity worldwide [1]. Susceptibility to CAD is influenced by combined effects of environmental and inherited genetic factors, and consequently some families are particularly affected [2]. In 2007, several genome-wide association studies consistently identified a region on chromosome 9p21.3 as being the most strongly associated with CAD [3], [4], [5], [6]. This finding has been replicated in multiple case-control studies in several population groups in numerous ethnicities [7], [8], [9], [10], [11], [12], [13], [14], [15], making 9p21.3 the most replicated molecular genetic association with coronary heart disease to date. Other variants at chromosome 9p21.3 have been linked with susceptibility to several other complex diseases including type 2 diabetes [16], [17], aortic aneurism [18], ischemic stroke [19], [20], several cancers [21], [22], [23], [24], [25], [26], [27], [28], [29] and frailty [30].

Within the 9p21.3 locus, multiple single nucleotide polymorphisms (SNPs) in strong linkage disequilibrium have been associated with CAD [3], [6], [31]. The risk (minor) allele occurs with high frequency among many populations (minor allele frequency ∼50% in European populations) [8], [10], [11], [12], [13], [14] and confers a modest, yet highly reproducible increase in risk of approximately 1.3-fold per copy [31]. It has been suggested that the 9p21.3 locus may have clinical utility as an early marker for CAD susceptibility [32].

The association between the 9p21.3 risk locus and CAD appears to be independent of established risk factors, including elevated lipid levels, high blood pressure, obesity and diabetes [5], [9], [12], and the mechanism underlying the association remains enigmatic. The risk locus contains no protein coding genes or known microRNAs. The nearest genes, approximately 100 kb upstream of the risk locus, are a pair of tumor suppressor genes (cyclin dependent kinase inhibitors, *CDKN2A* and *CDKN2B)* that are involved in regulation of the cell cycle and have no demonstrated role in CAD to date. The risk locus overlaps exons 13–20 of a recently identified large, non-coding, antisense RNA of unknown function, named *ANRIL* (antisense noncoding RNA in the INK4 locus, also known as *CDKN2BAS*) [33]. *ANRIL*, like other non-coding RNAs, is predicted to play a role in regulation of gene expression and is expressed in cells and tissues that are affected by atherosclerosis [9]. Variants associated with CAD are located within intronic and 3′ flanking sequences and recent data suggest that these, and other variants in close proximity, mediate risk of CAD by altering expression of *ANRIL* (and possibly decreasing expression of *CDKN2A* and *CDKN2B*) via multiple, independent *cis*-regulatory elements [34], [35] and, more specifically, by influencing the expression and structure of individual splice variants produced from the *ANRIL* gene [36], [37], [38], [39], [40]. The region contains a dense assembly of gene expression enhancers and two CAD risk SNPs are located in one of these motifs, which disrupts a binding site for the transcription factor, STAT1 [35]. Less clear are the risk allele-associated changes in expression of *CDKN2A* and *CDKN2B*; two reports find they are both concordantly decreased with *ANRIL* [38], [40], while decreased *CDKN2A* [34] and increased *CDKN2B* expression has also been reported [34], as well as strong evidence for direct involvement of *ANRIL* in epigenetic repression of both *CDKN2A* and *CDKN2B* [41], [42]. The downstream effects of altered activation of these regulators of the cell cycle progression pathway on CAD risk remain unknown.

We now have a greater understanding of the regulatory events at the genomic region proximal to the risk locus, but to understand the mechanism underlying the association between 9p21.3 and CAD, pathways involved in disease susceptibility in tissues critical to its pathogenesis need to be defined. Further downstream targets of *ANRIL* linking this genomic region to atherosclerotic processes fundamental to CAD have yet to be determined. We hypothesized that variants within the 9p21.3 risk locus may be associated with altered expression of genes in myocardial and vascular tissues, which contributes to the development of cardiovascular pathology. To test this hypothesis and identify pathways that might be influenced by the 9p21.3 variants, we investigated associations between rs1333049, a representative SNP from the 9p21.3 locus, with global gene expression in several key cardiovascular tissues, including heart tissue from donors with no previously diagnosed heart disease (predominant cause of death, cerebral vascular accident) and carotid plaque tissues from carotid endarterectomy patients. Our data suggest altered expression of multiple genes in these tissues and we propose a common transcriptional mechanism that might relate cardiovascular gene expression to the 9p21.3 risk locus.

## Results

### Clinical Characteristics and Genotype Frequencies in Heart Donors and Patients

The baseline characteristics of heart donors, heart valve patients and carotid endarterectomy patients are listed in Table 1. For all cohorts, the genotype frequencies were in Hardy-Weinberg equilibrium (donors p = 0.762, heart valve patients p = 0.701, carotid endarterectomy patients p = 1.00) and were in concordance with other European populations [6]. For heart donors, associations between baseline characteristics and 9p21.3 genotype are reported in Table 2.

**Table 1:** Baseline characteristics of heart donors, heart valve patients and carotid endarterectomy patients.

Variable		Heart Donors (n = 108)	Heart Valve Patients (n = 104)	Carotid Endarterectomy Patients (n = 106)
Age* (years)		48±13	62±10	70±9
Gender	Male (%)	55 (51)	78 (75)	85 (80)
	Female (%)	52 (48)	26 (25)	21 (20)
Ethnicity	European (%)	104 (96)	104 (100)	106 (100)
	African-American (%)	4 (4)	0 (0)	0 (0)
LVEF* (%)		52±15%	–	–
Cause of Death	Cerebral vascular accident (%)	78 (72)	–	–
	Gunshot wound (%)	10 (9)	–	–
	Motor vehicle accident (%)	9 (8)	–	–
	Head trauma (%)	7 (7)	–	–
	Other (%)	4 (4)	–	–
rs1333049	GG (%)	32 (30)	38 (37)	34 (32)
	GC (%)	55 (51)	48 (46)	52 (49)
	CC (%)	21 (19)	18 (17)	20 (19)

* mean±standard deviation.

**Table 2:** Baseline characteristics of heart donors by 9p21.3 (rs1333049) genotype and allele frequency.

Variable		rs1333049 genotype			allele		p-value(additive model)
		GG	GC	CC	G	C	
Age* (years)		45.0±13.9	49.4±12.3	48.2±11.2	–	–	0.280
Gender	Male (%)	18 (33)	27 (49)	10 (18)	0.57	0.43	0.519
	Female (%)	14 (27)	27 (52)	11 (21)	0.53	0.47	
Ethnicity	European	29 (28)	52 (51)	21 (21)	0.54	0.46	0.237
	A-A	2 (50)	2 (50)	0 (0)	0.75	0.25	
LVEF* (%)		45±16	57±14	49±14	–	–	0.329
Cause of Death	CVA (%)	19 (24)	42 (54)	17 (22)	0.51	0.49	0.230
	GW (%)	3 (30)	6 (60)	1 (10)	0.60	0.40	
	MVA (%)	4 (44)	4 (44)	1 (11)	0.67	0.33	
	HT (%)	3 (43)	2 (28)	2 (28)	0.57	0.43	
	Other (%)	3 (75)	1 (25)	0 (0)	0.88	0.12	

* mean±standard deviation.

A-A = African-American; CVA =  cerebral vascular accident; GW = gunshot wound; HT = head trauma; LVEF = left ventricular ejection fraction; MVA =  motor vehicle accident.

### Gene Expression Profile Associated with 9p21.3 Risk Allele in Myocardium

To investigate associations between 9p21.3 genotype and gene expression with minimal confounding from the influence of advanced coronary artery disease, genome-wide analysis of Affymetrix Human Gene 1.0 ST expression profiles was performed in cardiac tissue from heart donors. Despite having no prior diagnosis of a cardiac event, this group are likely to have a spectrum of subclinical atherosclerosis, consistent with their mean age of 48 years (Table 1). This analysis identified 59 gene transcripts that were putatively differentially expressed in association with the rs1333049 high-risk C allele (fold-change >1.1 per copy) at the chosen level of significance (p<0.05, not corrected for multiple comparisons). Of these, 46 transcripts remained significantly associated with 9p21.3 genotype after adjustment for age, gender, ethnicity and cause of death. The majority of these genes (70%) were down-regulated in association with the risk allele, and showed a modest fold-change in expression (median fold change  = 1.12 per copy of the risk allele, range 1.10–1.33). Analysis of the chromosomal location of the differentially expressed genes revealed an even distribution throughout the genome with no chromosome or cytoband significantly over-represented (Figure S1).

**Figure 1:**
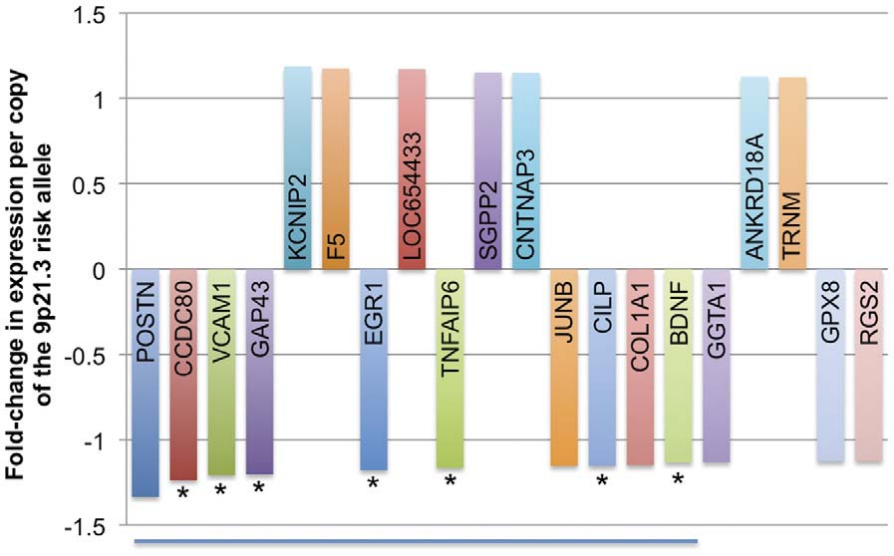
Twenty top-ranked genes altered in association with the 9p21.3 risk allele in donor hearts (n = 108). Analysis was performed using a fold-change threshold of >1.1 per copy of the risk allele and p<0.05 (adjusted for age, gender, ethnicity and cause of death; not corrected for multiple comparisons). Each bar represents an individual gene, as indicated by the gene symbol. Genes are ranked in order of fold-change from greatest to smallest (left to right). Analysis of the top 10 most down-regulated genes (indicated by line below graph) identified a shared combination transcription factor binding sites within the promoter regions of 7 of these genes (genes indicated by asterisks).

**Figure 2:**
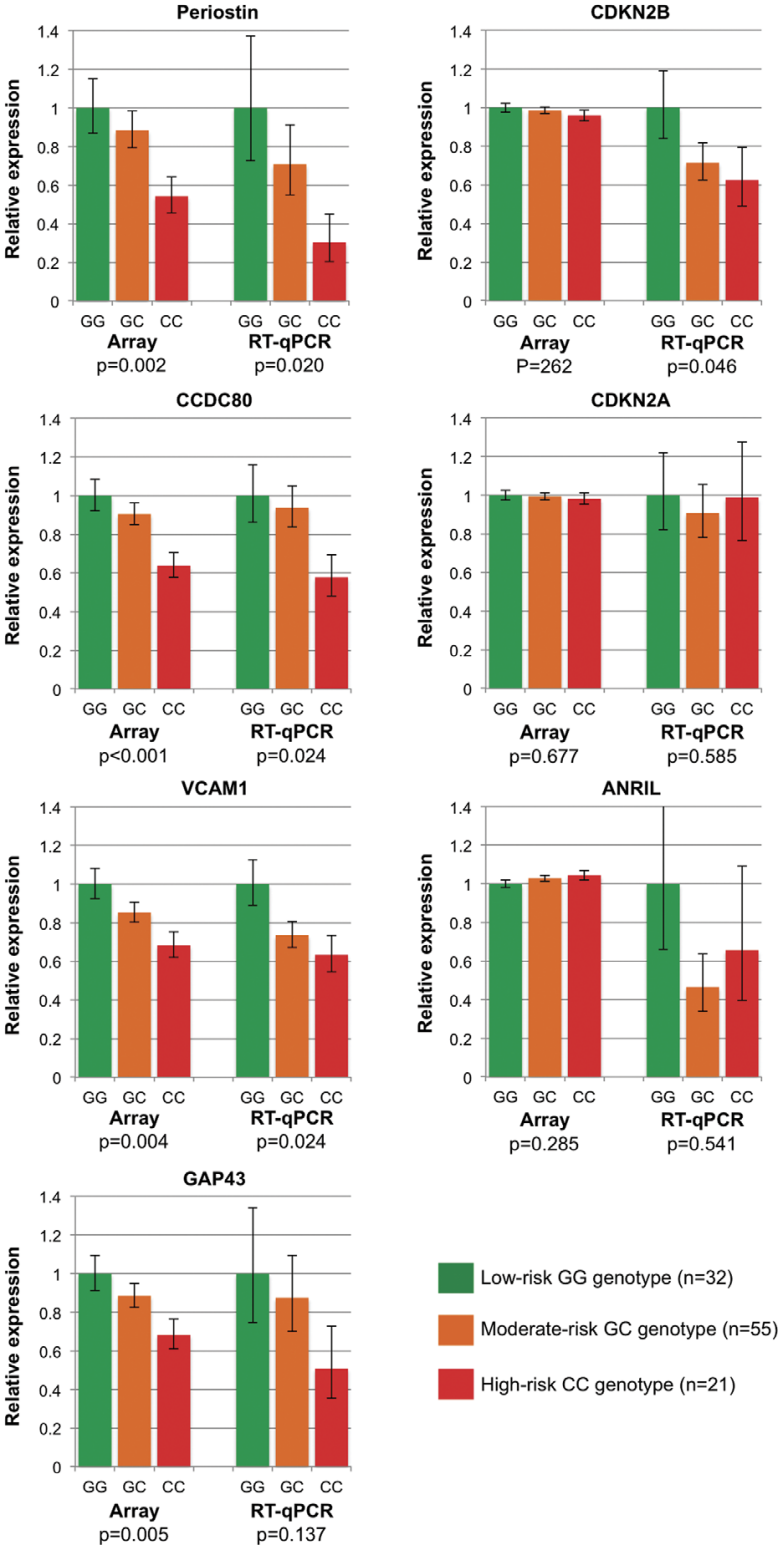
Comparison of microarray data with real-time PCR for selected genes in donor hearts (n = 108). Periostin, *CCDC80* (coiled-coil domain containing 80) and *VCAM1* (vascular cell adhesion molecule 1), the most differentially expressed genes altered in association with the 9p21.3 risk allele in heart donors, gave an equivalent, statistically significant decrease in expression by microarray and RT-qPCR methods. In contrast to array data, RT-qPCR for *CDKN2B*, a gene in close proximity to the 9p21.3 risk locus, also showed a significant decrease in expression. No significant association between the 9p21.3 risk allele and expression levels of *ANRIL* or *CDKN2A* was detected in these individuals, by either method. The 9p21.3 (rs1333049) low-risk GG genotype is depicted in green; GC heterozygotes (intermediate risk) are shown in orange; the high-risk CC genotype is shown in red. Statistical analysis was performed assuming an additive genetic model and all p-values have been adjusted for age, gender, ethnicity and cause of death.

The 20 most differentially expressed genes in heart donors are shown in Figure 1 (p<0.05 adjusted for age, gender, ethnicity and cause of death; additional information provided in Table S1). The gene encoding the extracellular matrix protein periostin, *POSTN*, was the most differentially expressed gene associated with rs1333049 genotype (down-regulated 1.33-fold per copy of the risk allele, p = 0.002). This was confirmed by RT-qPCR in the same samples, which showed that expression of *POSTN* was down-regulated 1.84-fold per copy of the risk allele (95% CI 1.10–3.06, p = 0.020, Figure 2) and that 5% of the variance in *POSTN* levels could be attributed to 9p21.3 genotype. Associations between 9p21.3 genotype and the expression of three other transcripts, *CCDC80*, *VCAM1* and *GAP43*, were also validated by RT-qPCR, although statistical significance was only replicated for *CCDC80* and *VCAM1* (p = 0.024 for both, Figure 2). While no associations between the risk allele and expression of genes in close proximity to the risk locus, including *ANRIL*, *CDKN2A*, *CDKN2B* and *MTAP,* were detected by array, RT-qPCR analysis showed lower expression of *CDKN2B* in individuals carrying the risk allele (down-regulated 1.35-fold per copy of the risk allele, p = 0.046, Figure 2). RT-qPCR corroborated array data for *CDKN2A* and *ANRIL*, which showed no significant difference between genotypes (*CDKN2A* p = 0.585; *ANRIL* p = 0.541, Figure 2). Overall, expression levels of *CDKN2B* and *CDKN2A*, and *CDKN2A* and *ANRIL* were positively correlated (Pearson correlation co-efficient *CDKN2B*/*CDKN2A* = 0.367, p = 0.001; *CDKN2A*/*ANRIL* = 0.252, p = 0.045). Expression of *ANRIL* and *POSTN* were also strongly positively correlated (Pearson correlation co-efficient 0.658, p = 4.59×10^−1−^).

Using a false discovery rate (FDR) of 0.001, analysis of Gene Ontology biological process (GO_biological process) terms associated with the differentially expressed transcripts identified significant enrichment for genes involved in response to wounding (p-value from gene set enrichment analysis (GSEA): 2.65×10^−10^), cell migration (p-value from GSEA: 4.89×10^−9^) and the inflammatory response (p-value from GSEA: 1.97×10^−7^), all processes that contribute to the development and progression of atherosclerosis. Consistent with these findings, analysis of proprietary GeneGo ontology terms (MetaCore database) revealed that the differentially expressed transcripts were enriched for gene biomarkers (genes previously reported to have altered expression in disease) of inflammation (p-value from GSEA: 7.28×10^−9^) and myocardial infarction (p-value from GSEA: 1.53×10^−9^), despite originating from cardiac tissue with no diagnosed heart disease (FDR = 0.001). The subset of genes associated with each of these GO_biological process and GeneGo ontology terms (25 genes in total) were predominantly down-regulated in association with the high-risk 9p21.3 allele (88%). Network analysis showed that these genes mapped to a set of receptors through direct interactions and formed part of a network of pathways that regulate a group of transcription factors, including NF-κB (p-value from network analysis: 1.25×10^−65^, Figure S2).

To investigate whether the differentially expressed genes may be co-regulated, the transcriptional control of the differentially expressed genes was analysed. This showed that the gene set was most strongly enriched by genes known to be regulated by the transcription factor Sp1 (18 out of 46 genes, p-value from network analysis: 4.83×10^−61^). Of the 10 most down-regulated genes, further analysis of the promoter regions revealed that specific combinations of transcription factor binding sites were over-represented among 7 of these genes: *CCDC80*, *VCAM1*, *GAP43*, *EGR1*, *TNFAIP6 CILP, BDNF* (Figure 1). Consistent with the network of genes associated with GO_biological process and GeneGo ontology terms above, these binding site signatures comprised a NF-κB binding site in close proximity (<200 bases) to an HMGB1 site (p-value from one-sided Fisher exact test: 1.14×10^−5^) and a RelA (NF-κB subunit) binding site in close proximity to a TATA binding protein site (6 genes, *CILP* not included; p-value from one-sided Fisher exact test: 2.11×10^−7^). Transcription factor binding sites were located in evolutionarily conserved regions of DNA that share more than 70% identity with the mouse genome. No combinations of transcription factor binding sites were identified among the promoter regions of the 10 most up-regulated genes in heart donors.

### Combined Analysis of Gene Expression Profiles Associated with the 9p21.3 Risk Allele Across Myocardial and Vascular Tissues

To identify common pathways associated with the 9p21.3 risk allele irrespective of differences in tissue type or disease state, an analysis of the genes most significantly differentially expressed in either myocardial, carotid plaque, aorta or mammary artery tissues was performed. An expression fold-change threshold of >1.10 per copy of the risk allele at a more conservative level of significance of p<0.01 (not corrected for multiple comparisons) was used for this analysis to reduce the potential confounding influence of the presence of coronary artery disease in patient samples and to obtain a feasible number of genes for canonical pathway modeling (computationally intensive). This analysis of expression profiles identified 59 gene transcripts in carotid plaque (56% down-regulated), 20 transcripts in aorta (media/intima, 60% down-regulated), 65 transcripts in mammary artery (media/intima, 60% down-regulated) and 11 transcripts in donor heart (73% down-regulated) tissues that were putatively differentially expressed in association with the high-risk rs1333049 C allele (154 unique transcripts in total, 60% down-regulated, Table S2). No transcripts were consistently altered in all tissues, although ubiquitin specific peptidase 15 (*USP15*), a gene shown to induce cardiac hypertrophy in mice [43], showed an equivalent increase in expression in aorta and mammary artery tissues (aorta +1.16-fold per copy of the risk allele, p = 0.008; mammary artery +1.15-fold, p = 0.003). No significant associations were detected between the risk allele and overall expression of genes in close proximity to the risk locus (*CDKN2A*, *CDKN2B*, *ANRIL*, *MTAP*) in any of the tissues (an expanded analysis of transcripts spanning a >10 Mb region around the risk locus, from *DMRTA1* to *IFNB1*, in heart donors is provided in Table S3).

**Figure 3:**
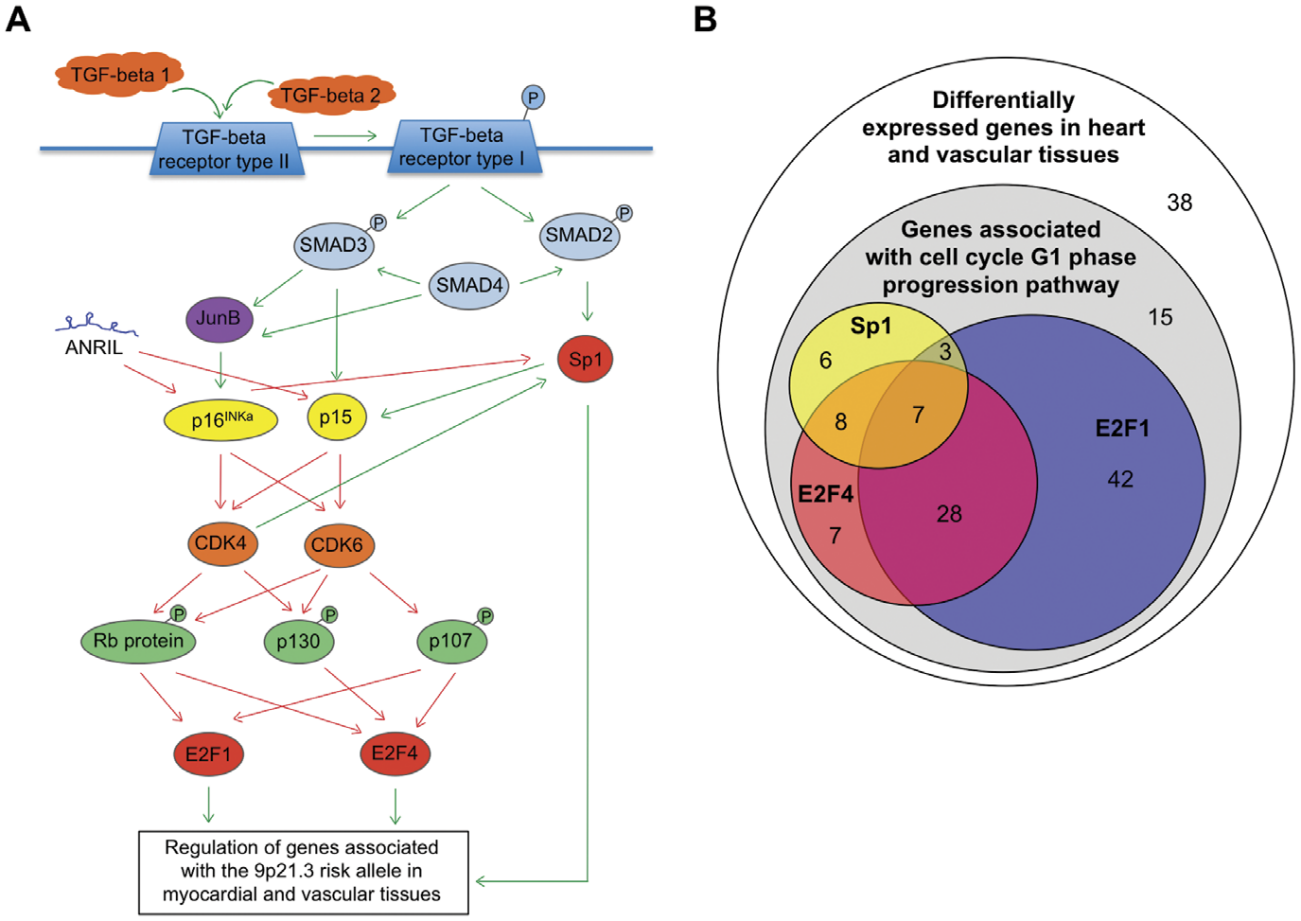
Canonical pathway modeling of the most significantly differentially expressed genes in donor heart (n = 108), carotid plaque (n = 106), aorta (n = 104) and mammary artery (n = 88) tissues. (A) A schematic depicting part of the cell cycle G1 phase progression pathway, indicating the regulatory role of p16^INK4^ and p15, which are encoded by two genes located adjacent to the 9p21.3 risk locus (*CDKN2A* and *CDKN2B)*. The majority of the most significantly differentially expressed genes (fold-change >1.1 per copy of the risk allele, p<0.01 not corrected for multiple comparisons,) associated with the 9p21.3 risk allele in myocardial and vascular tissues were predicted to be transcriptionally regulated by this pathway, predominantly by the transcription factors E2F1, E2F4 and Sp1 (116 out of 154 genes, see Table S2 for the list of genes associated with this pathway and Figure S2 for gene network analysis). More than half of these genes (58%) were down-regulated in association with the 9p21.3 risk allele, which would be concordant with lower levels of *ANRIL* expression. Green arrows indicate positive regulation; red arrows indicate negative regulation. (B) Venn diagram indicating the proportion of differentially expressed genes predicted to be regulated by E2F1, E2F4 and Sp1. The genes predicted to be regulated by each of these transcription factors is indicated by the symbols *, § and ¶ respectively, in Table S2.

Canonical pathway modelling of the genes most significantly altered in association with the 9p21.3 risk locus in myocardial and vascular tissues combined, identified a highly significant association with the cell cycle G1 phase progression pathway (p-value from canonical pathway modelling: 1.08×10^−258^, Figure 3), in which proteins encoded by *CDKN2A* and *CDKN2B* (p16 and p15, respectively) play an important regulatory role. Of the 116 out of 154 differentially expressed genes associated with this pathway, 58% were down-regulated in association with the 9p21.3 risk allele (Figure S3, Table S2). This analysis indicated that the differentially expressed genes were most likely to be transcriptionally regulated by this pathway, via transcription factors including E2F1, E2F4 and Sp1 (Figure 3). There were no combinations of transcription factor binding sites in common among the 10 most up- or down-regulated genes from each tissue. However, further analysis of the transcriptional regulation of the combined set of differentially expressed genes confirmed that they were most enriched for genes regulated by Sp1 (33 genes, p-value from network analysis: 6.70×10^−85^).

## Discussion

Using genome-wide expression profiling, we have identified genes whose expression patterns may be associated with the 9p21.3 CAD risk allele in donor myocardial tissue and in vascular tissues from CAD patients. Within heart, shared combinations of transcription factor binding sites among genes with putatively altered expression suggested co-regulation of these genes by a shared mechanism. Canonical pathway modeling of the most differentially expressed genes across all tissues identified the cell cycle G1 phase progression pathway as the most likely mechanism regulating their expression. This pathway facilitates the activation of genes required for cell cycle progression and cell proliferation [44] and is controlled by proteins encoded by *CDKN2A* and *CDKN2B*, two genes located adjacent to the 9p21.3 risk locus. Hence, we propose that risk variants at 9p21.3 increase the activation of this pathway in cardiovascular tissues, leading to a proliferative phenotype that promotes cardiac hypertrophy and vascular remodeling, and contributes to increased susceptibility to atherosclerosis.

We report significantly lower expression levels of *CDKN2B*, but not *CDKN2A* or *ANRIL*, in heart donors carrying the 9p21.3 risk allele. Recent studies suggest that genetic variation within the 9p21.3 region may alter the expression of *CDKN2B*, *CDKN2A*, *ANRIL* (the large non-coding RNA that spans the CAD risk locus), and/or other genes on chromosome 9 located up to 1 Mb from the risk locus [35], [36], [37], [38], [39], [40]. Consistent with these findings, lower protein levels of p15 (*CDKN2B*) and p16 (*CDKN2A*) have been reported in association with the 9p21.3 risk allele in aortic smooth muscle cells [45]. These effects may be mediated by disruption of enhancer elements within the risk locus [35] and/or by altering expression of *ANRIL* splice variants or overall transcript levels [35], [36], [37], [38], [39], [40]. These findings are concordant with the phenotype of the Chr4^Δ70kb/Δ70kb^ knock-out mouse, in which targeted deletion of the orthologous 9p21.3 risk interval on mouse chromosome 4 resulted in severely reduced expression of nearby genes, including *CDKN2A* and *CDKN2B*, and increased proliferation of vascular cells [46].

Our study analyzed genes from myocardial and vascular tissues that were putatively altered in association with the 9p21.3 risk allele to identify common pathways downstream of these modest effects that are relevant to CAD. We found that 75% of the most differentially expressed genes across all tissues, regardless of disease state, were predicted to be regulated by the pathway that controls the cell cycle transition from G1 to S phase (Figure 3). This pathway activates the E2F family of transcription factors, including E2F1 [47]. In addition to activating genes involved in cell proliferation, E2F1 has been shown to function as an inhibitory regulator for the inflammatory mediator NF-κB and down regulate cytokine-induced expression of adhesion molecules, including *VCAM1* [48]. Our data in heart tissue suggests down-regulation of multiple genes associated with inflammation and adhesion processes, including *VCAM1*. Furthermore, the shared transcription factor binding site signatures identified among conserved promoter regions of 7 of the top 10 most down-regulated genes, included either a Nf-κB or RelA (a subunit of NF-κB) binding site. Together, these results suggest that the cell cycle G1 phase progression pathway is activated in individuals with the 9p21.3 risk allele. This may promote a proliferative phenotype that leads to adverse cardiac hypertrophy and vascular remodeling, and an increased susceptibility to CAD. This is consistent with the findings of Jarinova *et al* [37], who reported altered expression of genes involved in cell proliferation in association with the 9p21.3 risk allele, in whole blood from healthy subjects and stable CAD patients. Activation of this pathway may be one of the mechanisms by which CAD risk variants may increase CAD risk.

The mechanism underlying increased activation of the cell cycle G1 phase progression pathway in individuals with the 9p21.3 risk allele may center on two genes located adjacent to the risk locus, *CDKN2A* and *CDKN2B*. These genes block this pathway by inhibiting the cyclin-dependent kinases (CDK) 4 and 6. Previous studies suggest that total expression levels of *CDKN2A*, *CDKN2B* and *ANRIL* are positively correlated in blood and tissues [34], [38], [40], with expression of *ANRIL* and one or both of *CDKN2A* and *CDKN2B* reported to be down-regulated in association with the risk allele [34], [37], [38], consistent with our study. In support of this, Harismendy *et al* [35] found that CAD risk SNPs altered the sequence of an enhancer element within the 9p21.3 risk locus and disrupted long-range physical interactions between the enhancer and the *CDKN2A/B* locus. To add another layer of complexity, expression of ANRIL splice variants may also be altered in individuals with the risk allele [36], [37], although splicing varies by tissue type [40]. In whole blood, Jarinova *et al.* [37] showed increased expression of short splice variants and decreased expression of a long splice variant of *ANRIL* association with the risk allele. Expression of the long *ANRIL* splice variant was correlated with expression of *CDKN2B*, suggesting that the shift from long to short splice variants may influence the expression of nearby genes. Although *ANRIL* has been shown to repress expression of *CDKN2A* and *CDKN2B* through recruitment and retention of polycomb repressive protein complexes at the 9p21.3 locus [41], [42], it is unknown whether short or other alternatively spliced *ANRIL* transcripts may increase the efficiency of this epigenetic mechanism. Further research is needed to determine whether protein levels of p16 (*CDKN2A*) and/or p15 (*CDKN2B*) are down-regulated in association with the 9p21.3 risk allele in tissues relevant to the development of CAD. This would be predicted to increase activation of the cell cycle G1 phase progression pathway and promote a proliferative phenotype.

It is unknown whether the modest putative changes in gene expression observed in the current study would be sufficient to promote the development of CAD in individuals with the 9p21.3 risk allele. Of particular relevance is the observation that deletion of the equivalent 9p21.3 risk region in mice did not cause CAD, despite a marked reduction in *CDKN2A* and *CDKN2B* expression and a proliferative phenotype [46]. This suggests that other factors may be required for the development of CAD and that activation of the cell cycle G1 phase progression pathway may represent just one aspect of the mechanism underlying the association between the 9p21.3 risk allele and increased CAD risk. Moreover, none of the genes identified as having altered expression in association with the 9p21.3 risk allele remained significant after correction for multiple comparisons and these findings need to be validated in an independent sample set. It should also be noted that the majority of heart donors were on life-support as a result of head trauma or cerebral vascular accident and thus the myocardial gene expression profile may have been affected by the traumatic injuries, acute drug treatments and underlying sub-clinical cardiac disease prior to the donation of tissue. Similarly, the gene expression profiles of vascular tissues were likely to have been affected by chronic drug treatment.

In summary, our data suggest that the 9p21.3 CAD risk locus may be associated with an altered pattern of gene expression in myocardial tissue from donors with no diagnosed heart disease and in vascular tissues from heart patients. These expression profiles, while tissue-specific, may be regulated through the cell cycle G1 phase progression pathway, which is inhibited by proteins encoded by *CDKN2A* and *CDKN2B,* two genes located adjacent to the risk locus. We speculate that the CAD risk variants decrease expression of *CDKN2A* and *CDKN2B*, which leads to activation of CDK4, CDK6, and downstream transcription factors, including E2F1, E2F4 and Sp1, and to altered expression of their target genes. Our data suggests that this network of genes may be altered in association with 9p21.3 risk SNPs in cardiovascular tissues, both before and after the onset of overt disease. These findings may help elucidate one of the mechanisms by which the 9p21.3 locus contributes to increased CAD risk.

## Methods

### Human Samples

#### Donor Heart Tissue

Heart tissue from the left ventricular free wall of organ donors was collected by the Cleveland Clinic Kaufman Center for Heart Failure human heart tissue bank (n = 108) between August 1993 - May 2005. Heart specimens were rapidly frozen in liquid nitrogen at the time of harvest and stored at −80°C until use. The decision that the heart could not be used for transplantation was made by members of the clinical organ procurement team. Reasons for rejection included histocompatibility mismatch, structural damage to the heart, cardiac disease or other elements of the medical history that made the donor undesirable. The research team was not contacted until donation for transplant had been ruled out and the family provided informed written consent to use the heart for research. The study was approved by the Cleveland Clinic Institutional Review Board (IRB 2378). All procedures were in accordance with institutional guidelines.

#### Aorta, Mammary Artery and Carotid Plaque Tissue

Tissue from the media/intima layers of aorta (n = 104) and mammary artery (n = 88) were collected from Swedish patients undergoing aortic valve surgery or surgery for aortic aneurysm and carotid plaque tissue was collected from 106 Swedish patients undergoing carotid endarterectomy as previously described [40]. All patients provided informed written consent. The study was approved by the Karolinkska University ethics committee (01–199, 02–146, 02–147, 2006/784–31/1, 2005/880–31/3) and all procedures were in accordance with institutional guidelines.

### Sample Preparation and Genotyping

#### Donor Heart Tissue

Total RNA and genomic DNA was simultaneously extracted from frozen tissue after automated grinding (Retsch Mixer Mill MM301, Haan, Germany) in TRIzol® (Invitrogen, Carlsbad, CA) and chloroform [49]. RNA was purified using RNeasy Midi columns (Qiagen, Valencia, CA) according to manufacturer’s instructions and tested for quantity and quality (by visual assessment of gel plots) with an Experion (Bio-Rad Laboratories, Hercules, CA). RNA samples were digested with DNase I (Invitrogen). First strand cDNA synthesis was performed from 2 µg of total RNA with Superscript III (Invitrogen), followed by RNase H digestion (Invitrogen) as previously described [50]. Genomic DNA was extracted from the remaining organic and interphase layers of the TRIzol® extract (see protocol at http://genome-www.stanford.edu/DFSP/materials.shtml). Individuals were genotyped in duplicate for rs1333049 with Taqman SNP assay C_1754666_10 (Applied Biosystems, Foster City, CA) on a Rotor-Gene 3000 and analyzed with Rotor-Gene version 6.1 software (Corbett Research, Sydney, Australia). Reactions were optimized for 10 µL volumes with 0.5× the recommended probe concentration. Genotypes were validated for a subset of randomly selected samples by re-genotyping (n = 66) or sequencing (n = 7, see Methods S1 and Table S4 for primer sequences). Both methods gave 100% concordance with original genotypes.

#### Aorta, Mammary Artery and Carotid Plaque Tissues

Total RNA was isolated from tissues and genomic DNA was isolated from peripheral blood leukocytes as previously described [40]. Genotyping for rs1333049 was performed for aorta and mammary artery samples with custom printed Cardiometabo chips (Illumina, San Diego, CA) on an iScan instrument (Illumina). Genotypes for the carotid plaque samples were imputed from Human 610W-Quad Bead array (Illumina) data [51].

### Microarrays

#### Donor Heart Tissue

Individual myocardial gene expression profiles were generated for all samples with Human Gene 1.0 ST arrays (Affymetrix, Santa Clara, CA) according to manufacturer’s instructions (GEO accession: GSE22253). These arrays use 25-mer oligonucleotides to measure mRNA transcript abundance and consist of 844,550 probes representing approximately 27,900 transcripts [52]. Probe intensities were estimated using linear models fitted to Robust Multi-Array (RMA)-background corrected and quantile normalized data with R software (http://www.R-project.org) and the Aroma.affymetrix (http://groups.google.com/group/aroma-affymetrix) and Bioconductor Limma packages [53], [54]. Transcripts were annotated using the file HuGene-1_0-st-v1.na25.hg18.transcript.csv created on 20 March 2008 available from http://www.affymetrix.com/support/technical/byproduct.affx? product = hugene-1_0-st-v1. Associations between rs1333049 genotype and myocardial gene expression were tested with R software and the Bioconductor Limma package [53], [54] using an additive genetic model (see Figure S4 for QQ plot). None of the associations remained significant after correcting for multiple comparisons using the method of Benjamini and Hochberg [55], and unadjusted p-values have been reported. Genes altered >1.1-fold per copy of the risk allele at an unadjusted p-value <0.05 that remained significant after adjusting for age, gender, ethnicity and cause of death (dichotomized as ‘cerebral vascular accident’ or ‘other’) were considered to be of potential biological significance. The conservative fold-change threshold (>1.1-fold per copy of the risk allele) was applied to limit bioinformatic analyses to those transcripts most likely to have a functional effect, however this may have resulted in some false-negative associations.

#### Aorta, Mammary Artery and Carotid Plaque Tissues

Individual gene expression profiles were generated for aorta and mammary artery samples with Human Exon 1.0 ST arrays (Affymetrix) and for carotid plaque samples with HG-U133 plus 2.0 arrays (Affymetrix, GEO accession: GSE21545) as previously described [51]. Gene array analysis of aorta, mammary artery and carotid plaque samples was performed using a additive genetic model, as previously described [40] (see Figure S4 for QQ plots).

### Real-Time PCR in Donor Heart Tissue

To validate array data in donor hearts, real-time PCR (RT-qPCR) was performed using Taqman gene expression assays with inventoried probes (Applied Biosystems) for *POSTN* (assay Hs00170815_m1), *CCDC80* (Hs00277341_m1), *VCAM1* (Hs01003372_m1) and *GAP43* (Hs00967138), the most differentially expressed genes putatively associated with rs1333049 genotype, and for *ANRIL* (Hs01390879_m1), *CDKN2A* (Hs00923894_m1) and *CDKN2B* (Hs00793225_m1), genes adjacent to the 9p21.3 risk locus. Reactions (20 µL or 10 µL) were performed in triplicate or duplicate on a 7500 Fast Real-Time PCR System (Applied Biosystems) or a Lightcycler 480 Real-Time PCR system (Roche Diagnostics, Indianapolis, IN) according to manufacturer’s instructions. Quantification was performed with 7500 software version 2.0 (Applied Biosystems) or Lightcycler 480 software release 1.5.0 (Roche). Expression levels were converted to relative quantities and normalized to signal recognition particle 14 (*SRP14*), tumour protein translationally controlled 1 (*TPT1*) and eukaryotic elongation factor 1A1 (*EEF1A1*), as previously described [56]. RT-qPCR gene expression data displayed consistently skewed distributions and were log-transformed prior to analysis. Associations between gene expression and 9p21.3 genotype were tested with linear regression adjusting for potential confounding factors (age, gender, ethnicity and cause of death dichotomized as ‘cerebral vascular accident’ or ‘other’) using SPSS Statistics software, version 19 (IBM). Data on post-mortem interval (the time between death and tissue collection) was unavailable and gene expression analyses were not adjusted for this factor. Relative expression levels of *POSTN*, *CCDC80*, *VCAM1*, *GAP43*, *CDKN2B*, *CDKN2A* and *ANRIL* were calculated from RT-qPCR and microarray data using geometric means and standard errors. No RT-qPCR analyses were performed in human vessel samples.

### Bioinformatic Analysis

#### Chromosomal Distribution of Differentially Expressed Genes

The chromosomal distribution of differentially expressed genes was analyzed using the Database for Annotation, Visualization and Integrated Discovery (DAVID) version 6.7 [57], [58] functional annotation tool and visualized using the R/Bioconductor package Geneplotter (http://www.bioconductor.org/packages/2.3/bioc/html/geneplotter.html) based on annotation from hugene10st.db (http://www.bioconductor.org/packages/2.3/data/annotation/html/hugene10st.db.html) originally sourced from Entrez Gene on April 2, 2008.

#### Gene Set Enrichment Analysis (GSEA)

The biological functions of the differentially expressed genes in heart donors was analyzed with Gene Ontology – biological process terms (GO_biological process, www.geneontology.org) and proprietary GeneGo ontology terms using the MetaCore database (GeneGo Inc, St Joseph, MI) functional enrichment by ontology tool. The MetaCore database (GeneGo Inc) includes a manually curated database of human protein-protein, protein-DNA and protein-compound interactions, metabolic and signaling pathways. GeneGo ontologies are represented by canonical pathway maps, cellular process networks, disease biomarker networks, drug target networks, toxicity networks and metabolic networks. The functional enrichment by ontology tool ranks the relevance of matches between the set of differentially expressed genes and GO_biological process and GeneGo ontology terms on the probability of the match occurring by chance, given the size of the database (p-value from GSEA). For all gene set enrichment analyses, the false discovery rate (FDR) was set at 0.001 to limit the number of false positive results to 0.1%.

#### Network Analysis

The relationship between the subset of differentially expressed genes in heart donors associated with GO_biological process and GeneGo ontology terms was analyzed with the MetaCore database (GeneGo Inc) analyze network (receptors) algorithm, using the default settings. Briefly, this algorithm generates a list of receptors and transcription factors closely associated with the differentially expressed genes (initial gene set) based on interactions contained within the MetaCore database, then builds a network for each receptor consisting of all the shortest paths (ie the smallest possible number of direct interactions) from the receptor to the nearest transcription factors. The probability that the intersection between the initial gene set and the genes/proteins in the network occurs by chance, given the size of the initial gene set, the network and the interaction database (p-value from network analysis), follows a hypergeometric distribution and was used to rank the relevance of the networks generated.

The association between individual transcription factors and differentially expressed genes in heart and vascular tissues was analyzed with the MetaCore database (GeneGo Inc) transcription regulation algorithm. This algorithm identifies the shortest path of direct interactions from the differentially expressed genes (the initial gene set) to the nearest transcription factors based on interactions in the MetaCore database, then builds a network for each transcription factor identified. The transcription factor networks were ranked on their statistical significance as described for the algorithm above.

#### Enrichment Analysis for Combinations of Transcription Factor Binding Sites (TFBS)

Promoter regions (−1500 bases to +200 bases from the transcription start site) of the top 10 most up- or down-regulated genes from each tissue were extracted and analyzed computationally for common transcription factor binding sites using oPOSSUM [59]. oPOSSUM determines the over-representation of transcription factor binding sites (TFBS) within a set of co-expressed genes compared with a pre-compiled background set. The background set comprises computationally predicted TFBS within evolutionarily conserved regions +/−10,000 bases of the predicted transcription factor start site in genes that are orthologous in human and mouse. To limit the number of false positive binding sites identified, the analysis was restricted to regions within the promoter that had >70% sequence identity between human and mouse and the transcription factor inter-binding distance was limited to a maximum of 200 base pairs. A one-tailed Fisher exact test was used to determine probability of a non-random association between the initial set of co-expressed genes and the combination of transcription factor binding sites identified.

#### Canonical Pathway Modelling

To identify pathways associated with the 9p21.3 risk allele across all tissues, canonical pathway modelling of the differentially expressed genes from all tissues combined was performed with the MetaCore database (GeneGo Inc) canonical pathway modelling algorithm, using the default settings. Genes altered >1.1-fold per copy of the risk allele at a p-value <0.01 (not corrected for multiple comparisons) from each tissue were considered to be of potential biological relevance and comprised the initial gene set for this analysis. A more stringent p-value threshold was selected to reduce the potential confounding influence of the presence of coronary artery disease in the patient samples and to obtain a feasible number of genes for canonical pathway modeling, which is computationally intensive. Briefly, this algorithm identifies all canonical pathways in the database that include genes in the initial gene set, then builds networks consisting of all genes/proteins and interactions from all such pathways. The networks were ranked on the probability that the intersection between the initial gene set and the genes/proteins in the network occured by chance, which is calculated using the hypergeometric probability distribution and takes into account the size of the initial gene set, the network and the number of genes/proteins associated with canonical pathways in the database (p-value from canonical pathway modelling).

## Supporting Information

Figure S1 **Chromosomal location of 46 transcripts identified as altered in association with the 9p21.3 risk allele (rs1333049) in the myocardium of donors.** Green bars indicate the location of transcripts that were up-regulated in association with the 9p21.3 risk allele; red bars indicate the location of transcripts that were down-regulated in association with the 9p21.3 risk allele (fold-change >1.1 per copy of the risk allele, p<0.05 adjusted for age, gender, ethnicity and cause of death; not corrected for multiple comparisons). The differentially expressed transcripts were not significantly clustered within individual chromosomes or within particular chromosomal regions.
(TIFF)

Figure S2 **Network analysis of 25 differentially expressed genes associated with gene ontology and MetaCore database biological process and biomarker terms in heart donors (n = 108).** These genes mapped to a single network that integrated several intracellular signalling pathways, potentially leading to regulation of multiple transcription factors. Most of the genes (70%) were down-regulated in association with the high-risk 9p21.3 allele (fold-change >1.1 per copy of the risk allele, p<0.05 adjusted for age, gender, ethnicity and cause of death; not corrected for multiple comparisons). The differentially expressed genes are indicated by red (up-regulated) or blue (down-regulated) circles adjacent to each gene, with the intensity of the colour representative of the magnitude of the fold-change in expression. Thick green lines indicate relationships between proteins that form part of canonical pathways.
(TIFF)

Figure S3 **Canonical pathway modeling of the most significantly differentially expressed genes in donor heart (n = 108), carotid plaque (n = 106), aorta (n = 104) and mammary artery (n = 88) tissues.** The majority of the most significantly differentially expressed genes (116 out of 154 genes, fold-change >1.1 per copy of the risk allele, p<0.01 not corrected for multiple comparisons) associated with the 9p21.3 risk allele in myocardial and vascular tissues were predicted to be transcriptionally regulated by this gene network, which forms part of the cell cycle G1 phase progression pathway. Of these genes, most were predicted to be regulated by the transcription factors E2F1, E2F4 and Sp1 (see Table S2 for the complete list of genes associated with this pathway, with those genes predicted to be regulated by E2F1, E2F4 and Sp1 indicated by the symbols *, § and ¶ respectively). The differentially expressed genes are indicated by red (up-regulated) or blue (down-regulated) circles adjacent to each gene, with the intensity of the colour representative of the magnitude of the fold-change in expression. Thick green lines indicate relationships between proteins that form part of canonical pathways.
(TIFF)

Figure S4 **QQ Plots for assessing the effect of 9p21.3 genotype on global gene expression in donor heart (n = 108), carotid plaque (n = 106), aorta (n = 104) and mammary artery (n = 88) tissues.** Each plot compares the distribution of p-values for all associations (y-axis) against a theoretical distribution of p-values assuming no effect (x-axis). For donor heart and vascular tissues, the observed distribution of p-values matched the theoretical null distribution or deviated below the line y = x, suggesting that any associations between gene expression and 9p21.3 genotype may have occurred by chance. Consequently, none of the associations remained significant after correction for multiple comparisons.
(TIFF)

Table S1 **The 20 most differentially expressed genes associated with the 9p21.3 risk allele in myocardium from 108 heart donors (fold-change >1.1 per copy of the risk allele, unadjusted p<0.05).**
(DOCX)

Table S2 **The most significantly differentially expressed genes associated with the 9p21.3 risk allele in donor myocardium, and patient aorta, mammary artery and carotid plaque tissues (fold-change >1.1 per copy of the risk allele, unadjusted p<0.01).**
(DOCX)

Table S3 **Affymetrix microarray analysis of associations between 9p21.3 (rs1333049) genotype and transcripts adjacent to the risk locus in heart donors.**
(DOCX)

Table S4 **PCR Primers for Sequencing rs1333049.**
(DOCX)

Methods S1 (DOCX)
