# Supplementary figures and images for: The Chromosome 9p21.3 Coronary Heart Disease Risk Allele Is Associated with Altered Gene Expression in Normal Heart and Vascular Tissues
Source: PLoS One. 2012 Jun 29;7(6):e39574. doi: 10.1371/journal.pone.0039574 (PMC3387158; doi:10.1371/journal.pone.0039574)

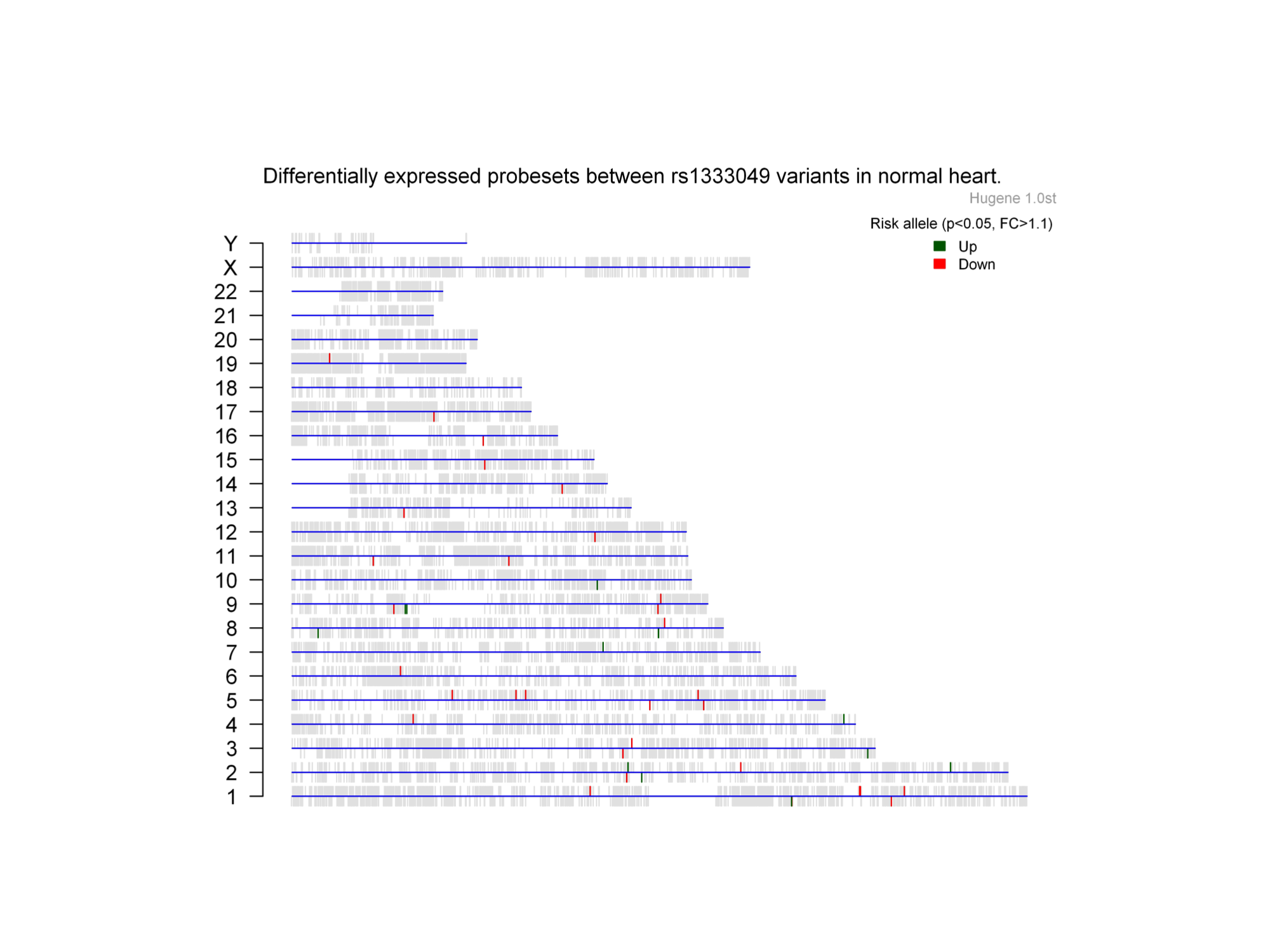

Supplement: Figure S1 — Chromosomal location of 46 transcripts identified as altered in association with the 9p21.3 risk allele (rs1333049) in the myocardium of donors. Green bars indicate the location of transcripts that were up-regulated in association with the 9p21.3 risk allele; red bars indicate the location of transcripts that were down-regulated in association with the 9p21.3 risk allele (fold-change >1.1 per copy of the risk allele, p<0.05 adjusted for age, gender, ethnicity and cause of death; not corrected for multiple comparisons). The differentially expressed transcripts were not significantly clustered within individual chromosomes or within particular chromosomal regions. (TIFF) [file pone.0039574.s001.tiff]

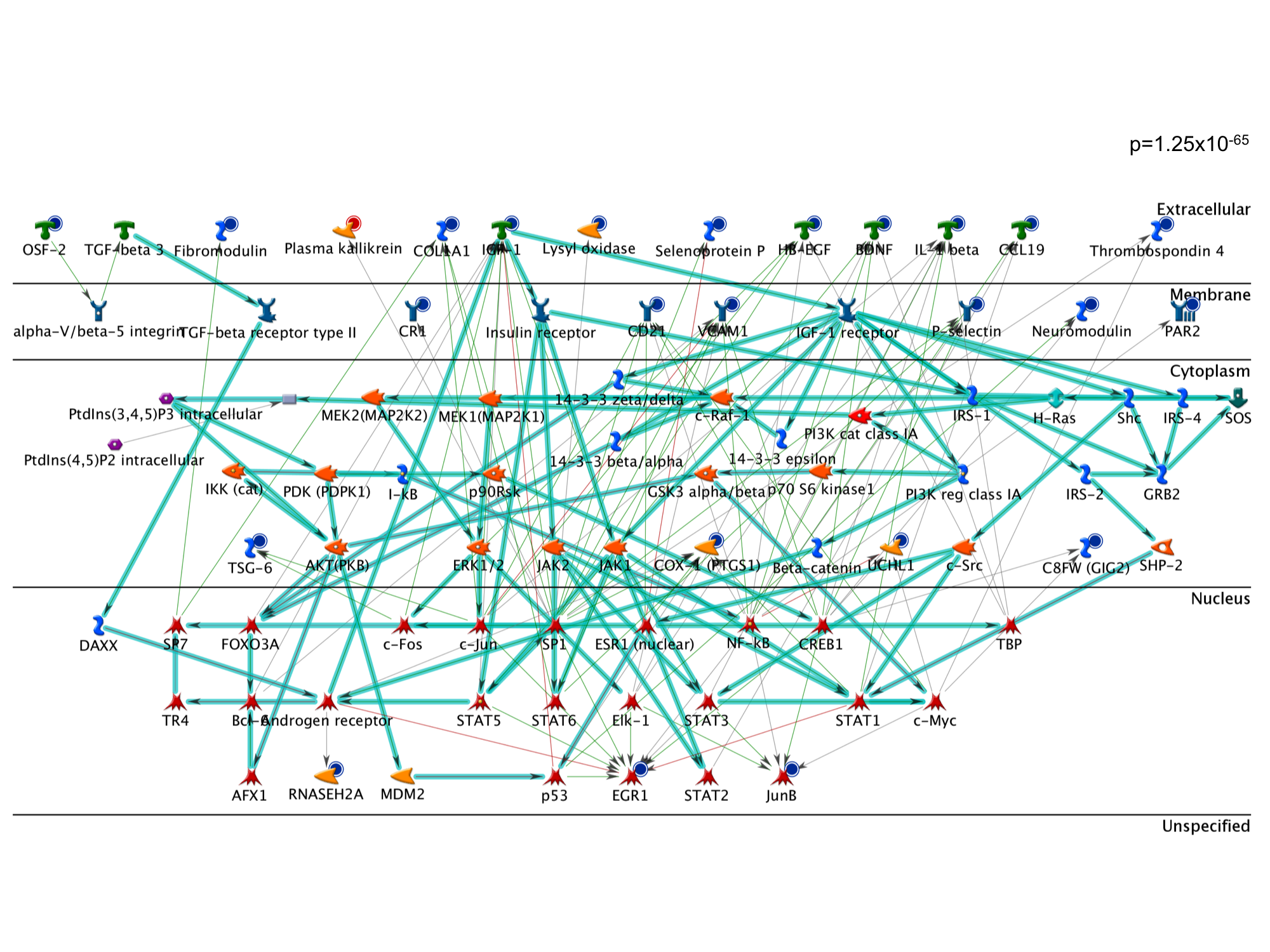

Supplement: Figure S2 — Network analysis of 25 differentially expressed genes associated with gene ontology and MetaCore database biological process and biomarker terms in heart donors (n = 108). These genes mapped to a single network that integrated several intracellular signalling pathways, potentially leading to regulation of multiple transcription factors. Most of the genes (70%) were down-regulated in association with the high-risk 9p21.3 allele (fold-change >1.1 per copy of the risk allele, p<0.05 adjusted for age, gender, ethnicity and cause of death; not corrected for multiple comparisons). The differentially expressed genes are indicated by red (up-regulated) or blue (down-regulated) circles adjacent to each gene, with the intensity of the colour representative of the magnitude of the fold-change in expression. Thick green lines indicate relationships between proteins that form part of canonical pathways. (TIFF) [file pone.0039574.s002.tiff]

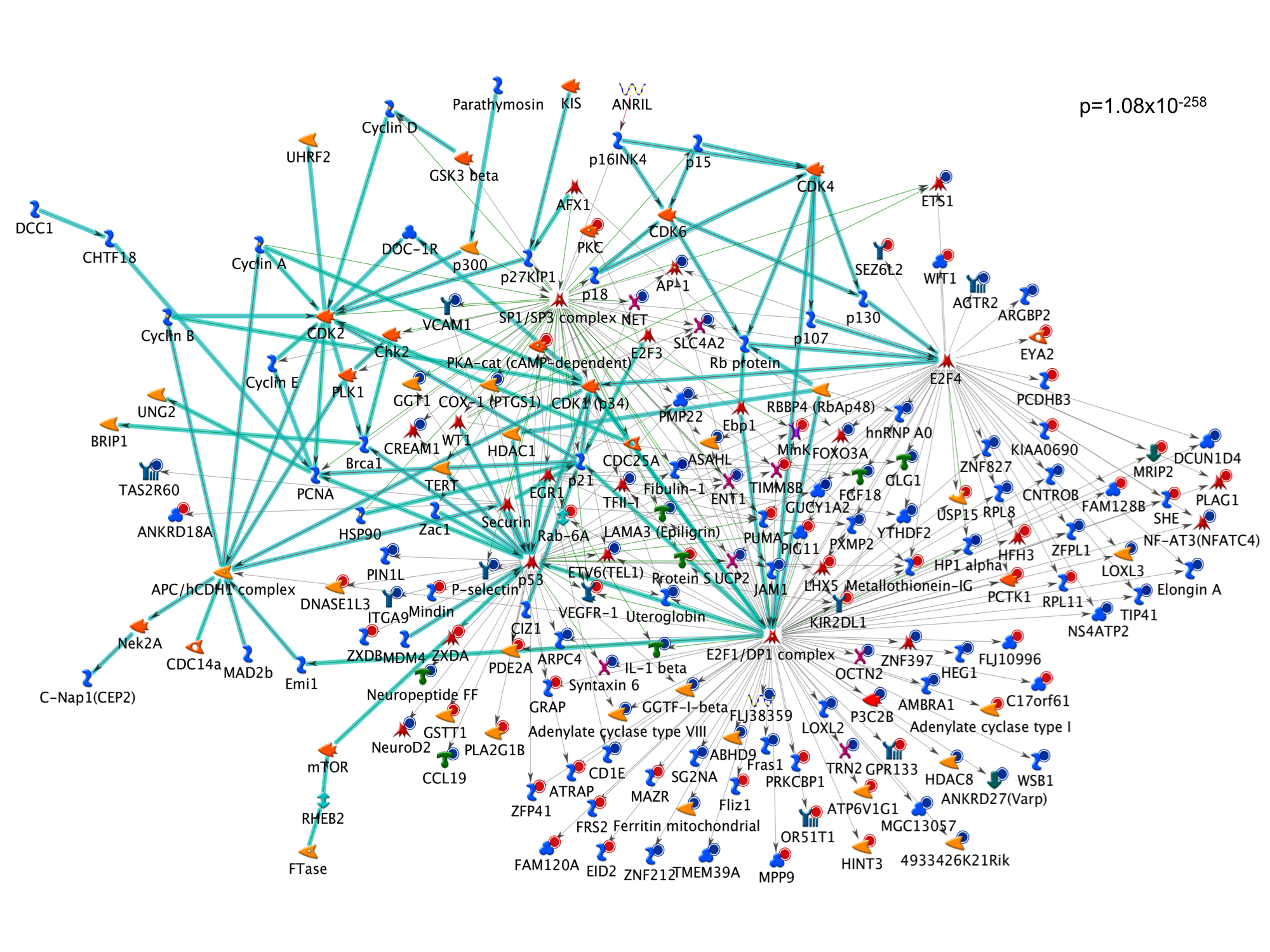

Supplement: Figure S3 — Canonical pathway modeling of the most significantly differentially expressed genes in donor heart (n = 108), carotid plaque (n = 106), aorta (n = 104) and mammary artery (n = 88) tissues. The majority of the most significantly differentially expressed genes (116 out of 154 genes, fold-change >1.1 per copy of the risk allele, p<0.01 not corrected for multiple comparisons) associated with the 9p21.3 risk allele in myocardial and vascular tissues were predicted to be transcriptionally regulated by this gene network, which forms part of the cell cycle G1 phase progression pathway. Of these genes, most were predicted to be regulated by the transcription factors E2F1, E2F4 and Sp1 (see Table S2 for the complete list of genes associated with this pathway, with those genes predicted to be regulated by E2F1, E2F4 and Sp1 indicated by the symbols *, § and ¶ respectively). The differentially expressed genes are indicated by red (up-regulated) or blue (down-regulated) circles adjacent to each gene, with the intensity of the colour representative of the magnitude of the fold-change in expression. Thick green lines indicate relationships between proteins that form part of canonical pathways. (TIFF) [file pone.0039574.s003.tiff]

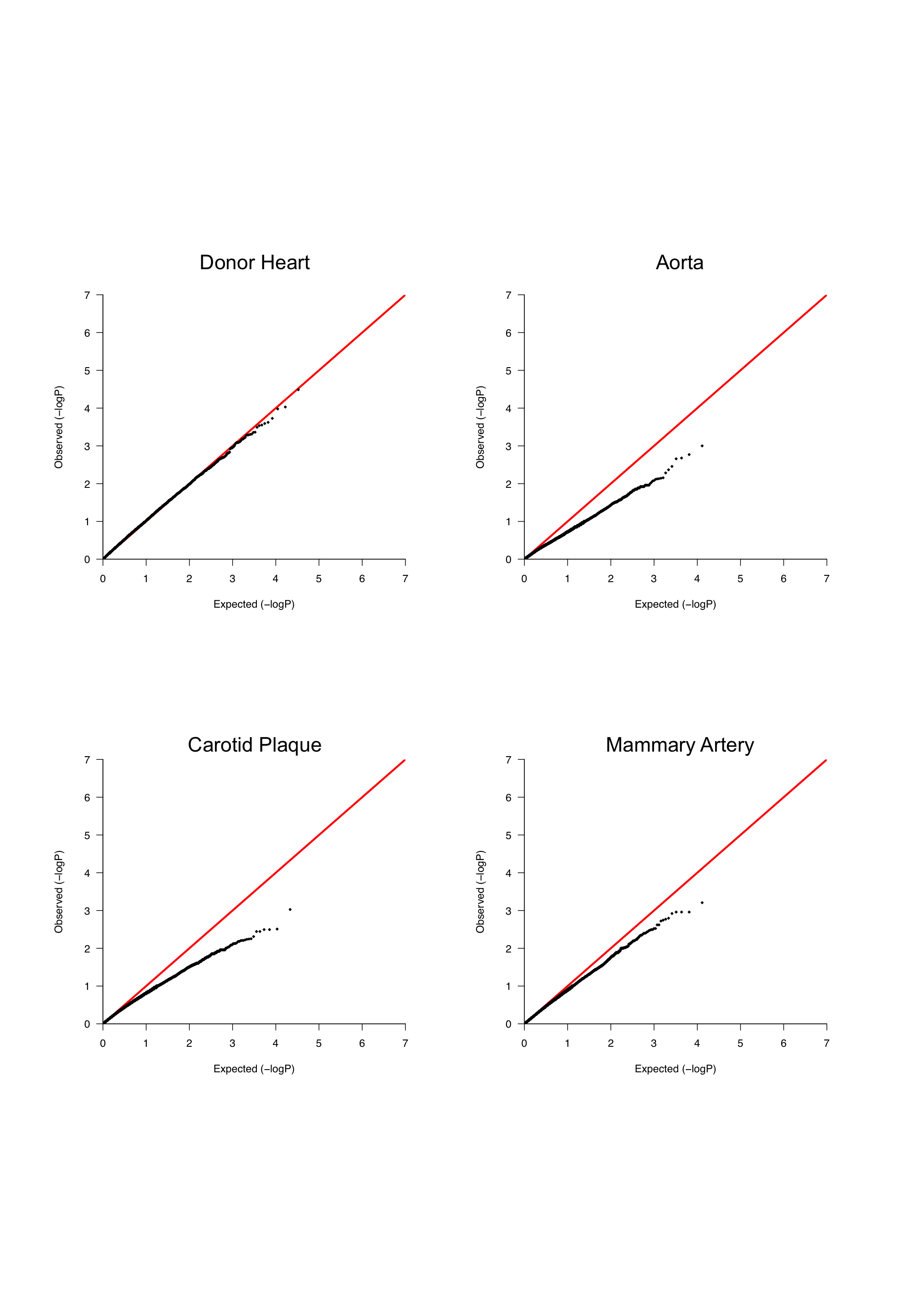

Supplement: Figure S4 — QQ Plots for assessing the effect of 9p21.3 genotype on global gene expression in donor heart (n = 108), carotid plaque (n = 106), aorta (n = 104) and mammary artery (n = 88) tissues. Each plot compares the distribution of p-values for all associations (y-axis) against a theoretical distribution of p-values assuming no effect (x-axis). For donor heart and vascular tissues, the observed distribution of p-values matched the theoretical null distribution or deviated below the line y = x, suggesting that any associations between gene expression and 9p21.3 genotype may have occurred by chance. Consequently, none of the associations remained significant after correction for multiple comparisons. (TIFF) [file pone.0039574.s004.tiff]
