# Supplementary material for: The Chromosome 9p21.3 Coronary Heart Disease Risk Allele Is Associated with Altered Gene Expression in Normal Heart and Vascular Tissues
Source: PLoS One. 2012 Jun 29;7(6):e39574. doi: 10.1371/journal.pone.0039574 (PMC3387158; doi:10.1371/journal.pone.0039574)
Supplement: Table S1 — The 20 most differentially expressed genes associated with the 9p21.3 risk allele in myocardium from 108 heart donors (fold-change >1.1 per copy of the risk allele, unadjusted p<0.05). (DOCX) [file pone.0039574.s005.docx]

**Supplementary Table 1. The 20 most differentially expressed genes associated with the 9p21.3 risk allele in myocardium from 108 heart donors (fold-change > 1.1 per copy of the risk allele, unadjusted p<0.05).**

| **Rank** | **Affymetrix Transcript #** | **Symbol** | **GeneTitle** | **Cytoband** | **Fold change** | **p-Value** | **p-Value*** |
| --- | --- | --- | --- | --- | --- | --- | --- |
| 1 | 7971077 | POSTN | periostin, osteoblast specific factor | 13q13.3 | -1.33 | 0.0096 | 0.0019 |
| 2 | 8089544 | CCDC80 | coiled-coil domain containing 80 | 3q13.2 | -1.23 | 0.0012 | 0.0003 |
| 3 | 7903358 | VCAM1 | vascular cell adhesion molecule 1 | 1p21.2 | -1.21 | 0.0023 | 0.0042 |
| 4 | 8081810 | GAP43 | growth associated protein 43 | 3q13.31 | -1.20 | 0.0106 | 0.0053 |
| 5 | 7935930 | KCNIP2 | Kv channel interacting protein 2 | 10q24.32 | 1.19 | 0.0306 | 0.0152 |
| 6 | 7922174 | F5 | coagulation factor V (proaccelerin, labile factor) | 1q24.2 | 1.18 | 0.0480 | 0.0453 |
| 7 | 8108370 | EGR1 | early growth response 1 | 5q31.2 | -1.18 | 0.0350 | 0.0413 |
| 8 | 8044605 | LOC654433 | hypothetical LOC654433 | 2q13 | 1.17 | 0.0124 | 0.0205 |
| 9 | 8045688 | TNFAIP6 | tumor necrosis factor, alpha-induced protein 6 | 2q23.3 | -1.16 | 0.0397 | 0.0381 |
| 10 | 8048717 | SGPP2 | sphingosine-1-phosphate phosphotase 2 | 2q36.1 | 1.15 | 0.0156 | 0.0077 |
| 11 | 8161460 | CNTNAP3 | contactin associated protein-like 3 | 9p13.1 | 1.15 | 0.0225 | 0.0305 |
| 12 | 8026047 | JUNB | jun B proto-oncogene | 19p13.2 | -1.15 | 0.0147 | 0.0328 |
| 13 | 7989750 | CILP | cartilage intermediate layer protein, nucleotide pyrophosphohydrolase | 15q22.31 | -1.15 | 0.0249 | 0.0118 |
| 14 | 8016646 | COL1A1 | collagen, type I, alpha 1 | 17q21.33 | -1.15 | 0.0302 | 0.0103 |
| 15 | 7947230 | BDNF | brain-derived neurotrophic factor | 11p13 | -1.13 | 0.0159 | 0.0157 |
| 16 | 8163908 | GGTA1P | glycoprotein, alpha-galactosyltransferase 1 pseudogene | 9q33.2 | -1.13 | 0.0437 | 0.0205 |
| 17 | 8161270 | ANKRD18A | ankyrin repeat domain 18A | 9p13.1 | 1.13 | 0.0002 | 0.0006 |
| 18 | 8165656 | TRNM | tRNA-Met | MT** | 1.13 | 0.0100 | 0.0247 |
| 19 | 8105348 | GPX8 | glutathione peroxidase 8 (putative) | 5q11.2 | -1.13 | 0.0190 | 0.0077 |
| 20 | 7908409 | RGS2 | regulator of G-protein signaling 2, 24kDa | 1q31 | -1.12 | 0.0255 | 0.0208 |

*adjusted for age, gender, ethnicity and cause of death

**mitochondrion
