# Supplementary material for: The Chromosome 9p21.3 Coronary Heart Disease Risk Allele Is Associated with Altered Gene Expression in Normal Heart and Vascular Tissues
Source: PLoS One. 2012 Jun 29;7(6):e39574. doi: 10.1371/journal.pone.0039574 (PMC3387158; doi:10.1371/journal.pone.0039574)
Supplement: Table S2 — The most significantly differentially expressed genes associated with the 9p21.3 risk allele in donor myocardium, and patient aorta, mammary artery and carotid plaque tissues (fold-change >1.1 per copy of the risk allele, unadjusted p<0.01). (DOCX) [file pone.0039574.s006.docx]

**Supplementary Table 2. The most significantly differentially expressed genes associated with the 9p21.3 risk allele in donor myocardium, and patient aorta, mammary artery and carotid plaque tissues (fold-change > 1.1 per copy of the risk allele, unadjusted p<0.01).**

|  | **Donor Myocardium** | | | **Carotid Plaque** | | | **Aorta** | | | **Mammary Artery** | | |
| --- | --- | --- | --- | --- | --- | --- | --- | --- | --- | --- | --- | --- |
| **Rank** | **Symbol** | **FC** | **p-value** | **Symbol** | **FC** | **p-value** | **Symbol** | **FC** | **p-value** | **Symbol** | **FC** | **p-value** |
| 1 | POSTN | -1.33 | 0.0096 | HINT3* | 1.35 | 0.0091 | GSTT1 | 1.43 | 0.0075 | FBLN1*§¶ | -1.43 | 0.0098 |
| 2 | CCDC80 | -1.23 | 0.0012 | CBX5*§ | -1.33 | 0.0032 | SPINK1 | -1.33 | 0.0085 | RNASE1 | -1.37 | 0.0032 |
| 3 | VCAM1¶ | -1.21 | 0.0023 | TIPRL*§ | -1.29 | 0.0074 | RNASE3 | -1.22 | 0.0045 | ADAM22 | -1.36 | 0.0013 |
| 4 | ANKRD18A | 1.13 | 0.0002 | RIMKLB* | -1.26 | 0.0093 | PCDHB3§ | 1.20 | 0.0039 | MT1G*§ | 1.35 | 0.0006 |
| 5 | SELP¶ | -1.12 | 0.0043 | SPON2 | 1.25 | 0.0060 | SLC4A2§¶ | -1.18 | 0.007 | GUCY1A2*§ | -1.35 | 0.0039 |
| 6 | CCDC93* | 1.11 | 0.0009 | LOC149832 | -1.24 | 0.0088 | USP15*§ | 1.16 | 0.0077 | PROS1*¶ | 1.29 | 0.0011 |
| 7 | PTGS1¶ | -1.11 | 0.0070 | SAP30L*§ | -1.23 | 0.0050 | WIT1§ | 1.13 | 0.0055 | GRAP* | 1.26 | 0.0100 |
| 8 | CCL19 | -1.11 | 0.0089 | ETV6*¶ | -1.23 | 0.0092 | EPHX3* | -1.13 | 0.0072 | JUN | -1.23 | 0.0096 |
| 9 | PPP1R3B | 1.11 | 0.0008 | FGF18*¶ | -1.22 | 0.0096 | SCGB1A1* | -1.12 | 0.001 | HEG1* | -1.22 | 0.0019 |
| 10 | IL1B* | -1.11 | 0.0064 | MPHOSPH9* | 1.22 | 0.0097 | RPL8*§ | -1.12 | 0.0022 | SEPN1 | 1.21 | 0.0018 |
| 11 | UCP2*§¶ | -1.10 | 0.0021 | ATP6V1G1* | 1.20 | 0.0062 | PATZ1* | 1.12 | 0.0033 | TIMM8B*§¶ | 1.21 | 0.0046 |
| 12 |  |  |  | SHE*§ | 1.20 | 0.0075 | AGAP4*§ | 1.12 | 0.0092 | KIR2DL1*§¶ | 1.2 | 0.0030 |
| 13 |  |  |  | FLT1*¶ | 1.19 | 0.0032 | ZC3H8* | 1.11 | 0.0035 | PLAG1*§ | 1.19 | 0.0015 |
| 14 |  |  |  | PDE2A* | 1.19 | 0.0062 | NEUROD2 | -1.11 | 0.0043 | OR4A5 | 1.19 | 0.0085 |
| 15 |  |  |  | PRKCZ | 1.18 | 0.0002 | OR1E2 | -1.11 | 0.0081 | C7orf67 | -1.18 | 0.0012 |
| 16 |  |  |  | WSB1* | -1.18 | 0.0046 | GGT1¶ | -1.11 | 0.009 | OR6C1 | 1.18 | 0.0012 |
| 17 |  |  |  | SEZ6L2§ | 1.18 | 0.0058 | FTMT* | -1.11 | 0.0093 | THNSL2 | -1.18 | 0.0024 |
| 18 |  |  |  | PRKACB | 1.17 | 0.0052 | C15orf53 | -1.1 | 0.005 | LHX5*§ | 1.18 | 0.0052 |
| 19 |  |  |  | TNPO2* | -1.17 | 0.0056 | GAFA1 | -1.1 | 0.0066 | FRAS1* | -1.17 | 0.0034 |
| 20 |  |  |  | RHOJ | 1.17 | 0.0097 | ELP1P | 1.1 | 0.0076 | OR9K2 | 1.15 | 0.0002 |
| 21 |  |  |  | SNORD123 | -1.16 | 0.0036 |  |  |  | ZXDA | 1.15 | 0.0016 |
| 22 |  |  |  | EYA2§ | 1.16 | 0.0066 |  |  |  | TR2IT1 | -1.15 | 0.0023 |
| 23 |  |  |  | GPR133* | 1.16 | 0.0067 |  |  |  | USP15*§ | 1.15 | 0.0033 |
| 24 |  |  |  | TP53I11*§ | 1.16 | 0.0088 |  |  |  | SLC29A1*§¶ | -1.15 | 0.0053 |
| 25 |  |  |  | FOXO3*§ | -1.15 | 0.0018 |  |  |  | THRSP | -1.15 | 0.0095 |
| 26 |  |  |  | ADCY1* | 1.15 | 0.0067 |  |  |  | CD1E* | -1.14 | 0.0007 |
| 27 |  |  |  | STRN3* | -1.15 | 0.0067 |  |  |  | GTF2IRD1¶ | -1.14 | 0.0038 |
| 28 |  |  |  | FAM120A* | 1.15 | 0.0068 |  |  |  | PIK3C2B* | -1.14 | 0.0041 |
| 29 |  |  |  | SLC6A2¶ | -1.15 | 0.0075 |  |  |  | OR51T1* | 1.14 | 0.0044 |
| 30 |  |  |  | CNTROB*§ | -1.15 | 0.0081 |  |  |  | C17orf61* | 1.14 | 0.0058 |
| 31 |  |  |  | MYO15B | 1.15 | 0.0095 |  |  |  | YTHDF2*§ | -1.14 | 0.0062 |
| 32 |  |  |  | HDAC8* | -1.14 | 0.0009 |  |  |  | ITGA9 | -1.14 | 0.0064 |
| 33 |  |  |  | FOXI1*§ | 1.14 | 0.0021 |  |  |  | PMP22§¶ | -1.14 | 0.0064 |
| 34 |  |  |  | ETS1§¶ | -1.14 | 0.0031 |  |  |  | RAB6A*¶ | 1.14 | 0.0094 |
| 35 |  |  |  | DCUN1D4*§ | -1.14 | 0.0041 |  |  |  | EID2* | 1.14 | 0.0098 |
| 36 |  |  |  | CAV2 | -1.14 | 0.0052 |  |  |  | ADCY3* | -1.13 | 0.0011 |
| 37 |  |  |  | PCTK1*§ | 1.14 | 0.0093 |  |  |  | C5orf4 | -1.13 | 0.0024 |
| 38 |  |  |  | DNASE1L3 | 1.13 | 0.0007 |  |  |  | NPFF | -1.13 | 0.0060 |
| 39 |  |  |  | TCEB3*§ | -1.13 | 0.0023 |  |  |  | FLJ43950 | -1.13 | 0.0068 |
| 40 |  |  |  | ZFP41* | 1.13 | 0.0057 |  |  |  | SLC15A2 | -1.13 | 0.0078 |
| 41 |  |  |  | HNRNPA0*§ | -1.13 | 0.0063 |  |  |  | GLG1*§ | -1.13 | 0.0095 |
| 42 |  |  |  | ZMYND8* | 1.13 | 0.0063 |  |  |  | RPL11*§ | 1.13 | 0.0096 |
| 43 |  |  |  | KIAA1522 | 1.13 | 0.0066 |  |  |  | LOC100129890 | 1.13 | 0.0097 |
| 44 |  |  |  | FRS2* | 1.13 | 0.0069 |  |  |  | KCNE1*¶ | 1.12 | 0.0030 |
| 45 |  |  |  | NFATC4*§ | -1.13 | 0.0083 |  |  |  | LAMA3*¶ | -1.12 | 0.0030 |
| 46 |  |  |  | ZFPL1*§ | -1.13 | 0.0090 |  |  |  | ANKRD27* | -1.12 | 0.0037 |
| 47 |  |  |  | TMEM39A* | -1.12 | 0.0004 |  |  |  | TAS2R60 | -1.12 | 0.0038 |
| 48 |  |  |  | FZR1 | -1.12 | 0.0025 |  |  |  | FAM128B§ | 1.12 | 0.0051 |
| 49 |  |  |  | LOXL2* | -1.12 | 0.0038 |  |  |  | PLA2G1B | 1.12 | 0.0058 |
| 50 |  |  |  | ZNF397* | -1.12 | 0.0045 |  |  |  | OK/SW-CL.58 | -1.12 | 0.0063 |
| 51 |  |  |  | LOC151009* | -1.12 | 0.0048 |  |  |  | C2orf88 | -1.12 | 0.0067 |
| 52 |  |  |  | SORBS2§ | -1.12 | 0.0058 |  |  |  | C5orf17 | 1.12 | 0.0076 |
| 53 |  |  |  | ARPC4* | -1.12 | 0.0076 |  |  |  | ZNF212* | -1.11 | 0.0032 |
| 54 |  |  |  | RPPH1 | -1.12 | 0.0090 |  |  |  | BSND | -1.11 | 0.0058 |
| 55 |  |  |  | STX6* | -1.11 | 0.0071 |  |  |  | NAAA*§¶ | -1.11 | 0.0059 |
| 56 |  |  |  | RRP12§ | 1.11 | 0.0094 |  |  |  | SIRPD | 1.11 | 0.0063 |
| 57 |  |  |  | AMBRA1* | -1.11 | 0.0096 |  |  |  | BBC3*§¶ | 1.11 | 0.0076 |
| 58 |  |  |  | hCG_1990547 | -1.1 | 0.0036 |  |  |  | PIN1L | -1.11 | 0.0076 |
| 59 |  |  |  | AGTRAP* | 1.1 | 0.0066 |  |  |  | F11R*¶ | -1.11 | 0.0077 |
| 60 |  |  |  |  |  |  |  |  |  | AGTR2§ | -1.1 | 0.0029 |
| 61 |  |  |  |  |  |  |  |  |  | SUSD1 | -1.1 | 0.0045 |
| 62 |  |  |  |  |  |  |  |  |  | SPINK5L2 | 1.1 | 0.0057 |
| 63 |  |  |  |  |  |  |  |  |  | ZNF827*§ | -1.1 | 0.0076 |
| 64 |  |  |  |  |  |  |  |  |  | SLC22A5* | -1.1 | 0.0087 |
| 65 |  |  |  |  |  |  |  |  |  | SLC43A3 | -1.1 | 0.0096 |

Red text indicates genes predicted to be regulated by the cell cycle G1 phase progression pathway (see Figure 3).

* indicates genes predicted to be regulated by the E2F1 transcription factor (85 genes)

§ indicates genes predicted to be regulated by the E2F4 transcription factor (45 genes)

¶ indicates genes predicted to be regulated by the Sp1 transcription factor (45 genes)
