# Supplementary material for: The Chromosome 9p21.3 Coronary Heart Disease Risk Allele Is Associated with Altered Gene Expression in Normal Heart and Vascular Tissues
Source: PLoS One. 2012 Jun 29;7(6):e39574. doi: 10.1371/journal.pone.0039574 (PMC3387158; doi:10.1371/journal.pone.0039574)
Supplement: Table S3 — Affymetrix microarray analysis of associations between 9p21.3 (rs1333049) genotype and transcripts adjacent to the risk locus in heart donors. (DOCX) [file pone.0039574.s007.docx]

**Supplementary Table 3. Affymetrix microarray analysis of associations between 9p21.3 (rs1333049) genotype and transcripts adjacent to the risk locus in heart donors.**

| **Transcript #** | **Gene Symbol** | **Fold-change** | **Direction** | **P-value** |
| --- | --- | --- | --- | --- |
| 8154656 | DMRTA1 | 1.02 | Up | 0.489 |
| 8154635 | MTAP/ANRIL | 1.03 | Up | 0.285 |
| 8160452 | CDKN2B | 1.03 | Down | 0.262 |
| 8160441 | CDKN2A | 1.01 | Down | 0.677 |
| 8160439 | MIR31 | 1.01 | Down | 0.773 |
| 8154627, 8160419 | IFNA1/IFNA13 | 1.02 | Down | 0.450 |
| 8154622 | IFNA8 | 1.00 | - | 0.931 |
| 8160422 | IFNA2 | 1.04 | Down | 0.193 |
| 8160417 | IFNA6 | 1.01 | Down | 0.829 |
| 8160405 | KLHL9 | 1.04 | Down | 0.076 |
| 8160401 | IFNA5 | 1.00 | - | 0.991 |
| 8160383, 8160377,  8160394 | IFNA14/IFNA7/IFNA4 IFNA10/IFNA17/ | 1.01 | Down | 0.708 |
| 8160392 | IFNA16 | 1.00 | - | 0.993 |
| 8160371 | IFNA21 | 1.03 | Down | 0.265 |
| 8160365 | IFNW1 | 1.03 | Up | 0.241 |
| 8160360 | IFNB1 | 1.00 | - | 0.936 |
