# Supplementary material for: The Chromosome 9p21.3 Coronary Heart Disease Risk Allele Is Associated with Altered Gene Expression in Normal Heart and Vascular Tissues
Source: PLoS One. 2012 Jun 29;7(6):e39574. doi: 10.1371/journal.pone.0039574 (PMC3387158; doi:10.1371/journal.pone.0039574)
Supplement: Table S4 — PCR Primers for Sequencing rs1333049. (DOCX) [file pone.0039574.s008.docx]

**Supplementary Table 4. PCR Primers for Sequencing rs1333049**

| **Primer** | **Sequence (5'-3')** | **Tm** |
| --- | --- | --- |
| Forward Primer  Reverse Primer | TGACCTTCATGCTATTTTGAGG  CTTGCTTACCTCTGCGAGTG | 59.2  58.8 |
