# Supplementary material for: The Chromosome 9p21.3 Coronary Heart Disease Risk Allele Is Associated with Altered Gene Expression in Normal Heart and Vascular Tissues
Source: PLoS One. 2012 Jun 29;7(6):e39574. doi: 10.1371/journal.pone.0039574 (PMC3387158; doi:10.1371/journal.pone.0039574)
Supplement: Methods S1 — (DOCX) [file pone.0039574.s009.docx]

**Methods**

Genotypes were validated for a subset of randomly selected samples by re-genotyping (n=66) or sequencing a fragment spanning rs1333049 (n=7) with a 3130*xl* Genetic Analyser (Applied Biosystems). Reactions (20μL) contained 1μL template DNA, 1x PCR buffer, 1.5mM MgCl_2_, 0.2 mM dNTPs (Fermentas, Glen Burnie, MD), 0.5mM forward and reverse primers (Supplementary Table 4) and 1U *Taq-Ti** DNA polymerase (Fisher Biotec, West Perth, Australia). The amplification profile comprised 2 mins polymerase activation at 94°C followed by 30 cycles of 94°C for 30s, 63°C for 30s, and 72°C for 30s, and 1 cycle of 72°C for 1min. Amplification gave a 216bp DNA fragment.
